# Development and validation of a nomogram for blood transfusion during intracranial aneurysm clamping surgery: a retrospective analysis

**DOI:** 10.1186/s12911-023-02157-9

**Published:** 2023-04-19

**Authors:** Shugen Xiao, Fan Liu, Liyuan Yu, Xiaopei Li, Xihong Ye, Xingrui Gong

**Affiliations:** Institute of Brain Disease and Neuroscience, Department of Anesthesiology, Xiangyang Central Hospital, Affiliated Hospital of Hubei University of Arts and Science, Xiangyang, Hubei China

**Keywords:** Blood transfusion, Hemoglobin, Intracranial aneurysm, Machine learning, Nomogram

## Abstract

**Purpose:**

Intraoperative blood transfusion is associated with adverse events. We aimed to establish a machine learning model to predict the probability of intraoperative blood transfusion during intracranial aneurysm surgery.

**Methods:**

Patients, who underwent intracranial aneurysm surgery in our hospital between January 2019 and December 2021 were enrolled. Four machine learning models were benchmarked and the best learning model was used to establish the nomogram, before conducting a discriminative assessment.

**Results:**

A total of 375 patients were included for analysis in this model, among whom 108 received an intraoperative blood transfusion during the intracranial aneurysm surgery. The least absolute shrinkage selection operator identified six preoperative relative factors: hemoglobin, platelet, D-dimer, sex, white blood cell, and aneurysm rupture before surgery. Performance evaluation of the classification error demonstrated the following: K-nearest neighbor, 0.2903; logistic regression, 0.2290; ranger, 0.2518; and extremely gradient boosting model, 0.2632. A nomogram based on a logistic regression algorithm was established using the above six parameters. The AUC values of the nomogram were 0.828 (0.775, 0.881) and 0.796 (0.710, 0.882) in the development and validation groups, respectively.

**Conclusions:**

Machine learning algorithms present a good performance evaluation of intraoperative blood transfusion. The nomogram established using a logistic regression algorithm showed a good discriminative ability to predict intraoperative blood transfusion during aneurysm surgery.

## Introduction

Blood transfusion is very common in patients undergoing major surgery. Blood transfusion can save lives by increasing tissue perfusion and oxygen delivery in patients with massive blood loss but can also result in transfusion-related adverse events, including fever, infection, acute lung injury, coagulation disorder, and immune dysfunctions [1–3]. Previous studies have demonstrated that transfusion is an independent relative factor for perioperative complications in patients undergoing cerebral aneurysm surgery [4], while angiography has confirmed the presence of vasospasm after blood transfusion [5]. Consequently, early identification of patients who require blood transfusion before surgery is both useful and necessary for preoperative preparation and implementing preventive strategies.

Traditionally, the serum hemoglobin (HB) level is the most frequently used indicator for blood transfusion [6]. Guidelines recommend that patients should receive transfusion in the setting of profound anemia [7, 8]. However, the guidelines are limited as they do not refer to other perioperative risk factors including age, sex, disease severity, procedure type, and pre-existing co-morbidity [9]. For example, while an HB of 80 g/L is acceptable for a young adult, it is not for a geriatric patient with ischemic coronary artery disease. Thus, HB should not be the only indicator for blood transfusion. However, an effective formula to predict the risk of transfusion is still lacking. In recent years, machine learning methods have been widely used to establish robust predictive models in the perioperative period, often without pitfalls and restrictions [10, 11]. such prediction models could help physicians to acutely identify the need for intraoperative blood transfusion preoperatively and enhance patient safety, reduce costs, and avoid transfusion-related complications. Thus, in the current study, we aimed to establish a nomogram to predict the probability of intraoperative blood transfusion in patients undergoing aneurysm surgery.

## Materials and methods

### Ethics statement and patient selection

The study was performed in adherence with the Declaration of Helsinki and its later amendments. The study was approved by the Ethics Committee of Xiangyang Central Hospital, affiliated with Hubei University of Arts and Science, and the requirement for written informed consent was waived, and the personal identifiers were removed before the data analysis.

We retrospectively scrutinized a total of 390 patients who underwent intracranial aneurysm clipping surgery in our hospital from January 2019 to December 2021. Variables with more than 20% missing values or less than 10 cases were eliminated. Finally, seven patients were excluded for previous intracranial surgery at the same site and eight patients were excluded due to taking oral anticoagulants before surgery. Therefore, a total of 375 patients who underwent intracranial aneurysm clipping surgery were included in the analysis. The aneurysm diagnosis was insured using cerebral angiography. Blood transfusion was defined as receiving packed red blood cells intraoperatively. Intraoperative transfusion was conducted when the HB level was less than 70 g/L in stable patients and less than 90 g/L in patients with unstable hemodynamics. The decision for blood transfusion was discussed between the anesthetist and surgeon.

### Risk factors

We collected and analyzed the following factors of the subjects: general information (sex, age, weight, previous cerebral disease, diabetes, cardiovascular disease, pulmonary disease, renal disease, liver disease, and diabetes mellitus [DM]), characteristics of the aneurysm (one or multi-site aneurysm, and whether it ruptured before surgery), characteristics of the patient status (American Society of Anesthesiologists [ASA], and heart function), and laboratory test results (white blood cell [WBC], HB, platelet [PLT], thromboplastin time [PT], activated partial thromboplastin time [APTT], fibrinogen, D-dimer, total protein [TP], and albumin [ALB]), intraoperative blood transfusion volume and vasopressor utility, postoperative mechanical ventilation duration, and length of postoperative hospital stay.

### Statistical analysis

#### Data imputation, standardization, and feature selection

**Fig. 1:**
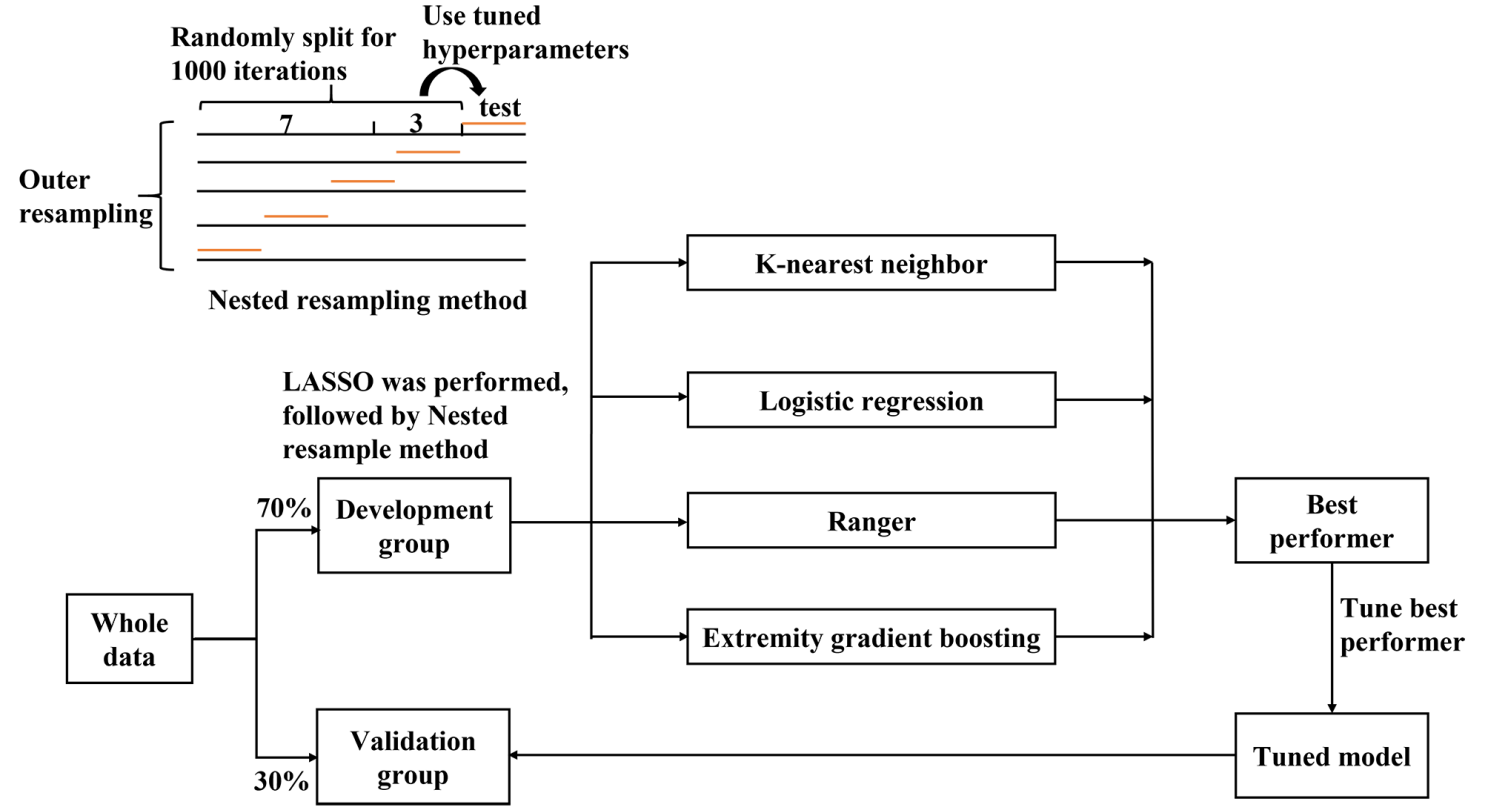
Flow chart of the trial. All data were split into the development and validation groups at a ratio of 7:3. The machine learning methods were performed with the training group data using a nested resampling method, in which the inner loop data were split at a 7:3 ratio with 1000 iterations to result in the best hyperparameter. Then, the best hyperparameters were used to train the machine model and tested in outer loop data with a 5-fold cross validation (CV), which resulted in a balanced performance evaluation. The machine learning that had the best performance evaluation was selected to establish the prediction model with the development data, before evaluating the prediction model with the validation data

First, the entire data were split into the development and validation groups at a ratio of 7:3 (Fig. 1). The missing data were imputed using recursive partitioning and the regression trees method with 10-fold cross-validation, followed by standardization of the variables to the same range of values with the max-min method before modeling in the development and validation groups [12]. The least absolute shrinkage and selection operator (LASSO; glmnet package in R with α = 1) classifier with 10-fold cross-validation was used to reduce the data set to its most meaningful features [13]. LASSO shrinks the coefficients, and those features with a coefficient of zero were excluded from the model establishment process. Variables entered before λ1se were chosen for the model establishment and evaluation process.

#### Machine learning algorithms

Four algorithms with nested resample methods were applied to build models to predict intraoperative blood transfusion, namely the K-nearest neighbor algorithm (KNN), logistic regression model (LR), ranger (also called random forest), and extremely gradient boosting machine (xgboost) (Fig. 1). The KNN, which is based on analogical reasoning, stores all of the training data and classifies the new data point based on similarity measures [14]. Ranger and xgboost are tree-based ensemble algorithms. Ranger generates multiple decision tree models by bootstrap samples and makes decisions through averaging or majority voting [12]. Xgboost builds a regression or classification tree model from the beginning and constructs the new models to maximally reduce the negative gradient of the loss function [15].

#### Machine learning algorithm selection

The machine learning tuning process and performance evaluation used a nested resampling method with the development data. Briefly, in the inner loop, all of the possible combinations of parameters were tried to obtain optimal hyper-parameters, and then the best hyperparameters were used to train the machine model before the model performance was evaluated in the outer loop. The inner loop used a holdout cross-validation (CV; ratio: 7:3) with 1000 iterations, and the outer loop used a 5-fold CV, which results in a balanced performance evaluation. We selected classification error (CE = (false negative + false positive)/(true negative + true positive + false negative + false positive)) to evaluate the model performance.

#### Nomogram development and assessment

The machine learning model with the best performance was chosen for nomogram establishment and assessment. We then established a nomogram of intraoperative blood transfusion using the development data and assessed the discriminative ability in both the development and validation sets.

The quantitative data were expressed as the mean and standard division (SD) if they were normally distributed, and the count data were expressed as numbers and proportions. The quantitative data were analyzed using the unpaired *t-test*, and the count data were analyzed using the χ2 test. The discriminative ability of the nomogram for predicting a blood transfusion was assessed using the area under the curve (AUC) of the receiver operating characteristic (ROC) curve. Statistical analysis was performed using R software (version 4.2.1). A *P* < 0.05 indicated the statistical difference.

## Results

### Patient demographics

The patient baseline characteristics are shown in Table 1. A total of 375 patients were enrolled for analysis, among whom 108 (25 man and 83 women, *P* < 0.001) patients received a blood transfusion. More patients who had ruptured aneurysms before surgery received blood transfusions compared with non-ruptured patients (85/23 vs. 143/124, *P* < 0.001). There were no significant differences between the transfused and non-transfused groups in terms of a previous history of cerebral disease, cardiovascular disease, pulmonary disease, renal disease, and DM (*P* > 0.05). Patients with ASA III-V received blood transfusions more frequently compared with those with ASA I-II (*P* < 0.001), while heart function did not affect blood transfusion (*P* > 0.05). Patients who received blood transfusion had lower levels of HB and PLT and a higher level of D-dimer than those who did not receive a blood transfusion (*P* < 0.001). Laboratory results of WBC, PT, APTT, fibrinogen, TP, and ALB were not significantly different between the transfusion and non-transfused groups before surgery (P > 0.05). Patients who received blood transfusion had a higher incidence of vasopressor utility and longer postoperative mechanical ventilation duration than those who did not receive a blood transfusion (P < 0.05), while the length of postoperative hospital stay was not significantly different between the two groups (P > 0.05).

**Table 1:** Patients baseline characteristics

	Total	Non-transfused	Transfused	P-value
**Sex**				< 0.001
Man	141 (37.6%)	116 (82.3%)	25 (17.7%)	
Woman	234 (62.4%)	151 (64.5%)	83 (35.5%)	
**Age (Mean (SD))**	60.4 (± 9.3)	60.3 (± 9.0)	60.7 (± 9.9)	0.54
**Weight (Mean (SD))**	63.7 (± 9.7)	64.1 (± 9.8)	62.8 (± 9.5)	0.13
**Multi-site**				0.85
One-site	337 (89.9%)	239 (70.9%)	98 (29.1%)	
Multi-site	38 (10.1%)	28 (73.7%)	10 (26.3%)	
**Ruptured**				< 0.001
None	147 (39.2%)	124 (84.4%)	23 (15.6%)	
Ruptured	228 (60.8%)	143 (62.7%)	85 (37.3%)	
**Cerebral-disease**				0.54
None	118 (31.5%)	87 (73.7%)	31 (26.3%)	
Present	257 (68.5%)	180 (70.0%)	77 (30.0%)	
**Cardiovascular-disease**				0.41
None	359 (95.7%)	257 (71.6%)	102 (28.4%)	
Present	16 (4.3%)	10 (62.5%)	6 (37.5%)	
**Pulmonary-disease**				0.52
None	347 (92.5%)	245 (70.6%)	102 (29.4%)	
Present	28 (7.5%)	22 (78.6%)	6 (21.4%)	
**Renal-disease**				0.44
None	356 (94.9%)	255 (71.6%)	101 (28.4%)	
Present	19 (5.1%)	12 (63.2%)	7 (36.8%)	
**DM**				0.3
None	357 (95.2%)	252 (70.6%)	105 (29.4%)	
Present	18 (4.8%)	15 (83.3%)	3 (16.7%)	
**ASA**				0.029
I~II	60 (16.0%)	50 (83.3%)	10 (16.7%)	
III~V	315 (84.0%)	217 (68.9%)	98 (31.1%)	
**Heart-function**				0.68
I~II	369 (98.4%)	262 (71.0%)	107 (29.0%)	
III~IV	6 (1.6%)	5 (83.3%)	1 (16.7%)	
**WBC (Mean (SD))**	11.2 (± 4.6)	10.9 (± 4.5)	11.8 (± 4.9)	0.064
**HB (Mean (SD))**	129.8 (± 17.2)	134.0 (± 15.4)	119.8 (± 17.3)	< 0.001
**PLT (Mean (SD))**	206.8 (± 60.0)	218.2 (± 59.5)	178.9 (± 51.9)	< 0.001
**PT (Mean (SD))**	13.4 (± 0.8)	13.4 (± 0.9)	13.5 (± 0.7)	0.18
**APTT (Mean (SD))**	33.2 (± 3.9)	33.3 (± 4.0)	33.1 (± 3.8)	0.58
**Fibrinogen (Mean (SD))**	3.3 (± 1.0)	3.2 (± 0.9)	3.4 (± 1.1)	0.55
**D-dimer (Mean (SD))**	2.5 (± 3.4)	2.2 (± 3.1)	3.4 (± 4.0)	< 0.001
**TP Mean (Mean (SD))**	67.5 (± 7.2)	67.6 (± 7.1)	67.4 (± 7.6)	0.74
**ALB Mean (Mean (SD))**	41.9 (± 4.0)	42.1 (± 3.6)	41.4 (± 4.7)	0.52
**Vasopressors**				
none	198 (52.8%)	153 (77.3%)	45 (22.7%)	0.006
present	177 (47.2%)	114 (64.4%)	63 (35.6%)	
**Blood transfusion (mL) (median [IQR])**	0.00 [0.00, 600.00]	0.00 [0.00, 0.00]	1100 [800, 1250]	< 0.001
**Ventilation duration (h) (median [IQR])**	0.00 [0.00, 12.00]	0.00 [0.00, 6.00]	9.00 [0.00, 24.00]	< 0.001
**Postoperative hospital stay (median [IQR])**	16.00 [12.00, 22.00]	16.00 [12.00, 21.00]	18.00 [13.00, 23.75]	0.104

Results are expressed as mean (SD) for continuous data and n (proportion) for categorical data. APTT, Activated partial thromboplastin time; ASA, American Society of Anesthesiologists; DM, Diabetes mellitus; HB, Hemoglobin; PLT, Platelet; PT, Prothrombin time; TP, Total protein; ALB, Albumin; WBC, White blood cell

### Feature selection and machine learning model performance evaluation

**Fig. 2:**
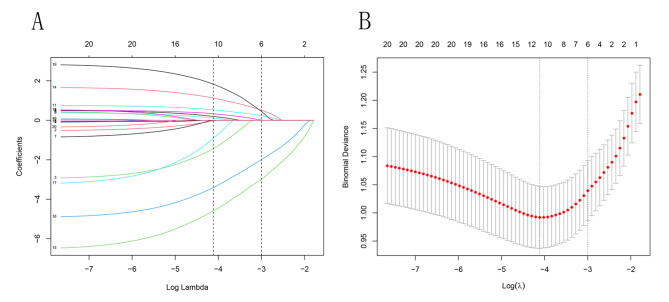
Results of the least absolute shrinkage and selection operator analysis of all data are shown in (**A**) and (**B**). (**A**) The different values of λ are shown on the x-axis, where each line represents one of the explanatory variables and its role in the model. The plots demonstrate that to what extent variables that enter the model influence the response variable. A variable that enters the model earlier influences the model more than that enters the model later. (**B**) The different values of λ are shown on the x-axis, while the binary deviance is shown on the y-axis. λ min to 1se indicates the acceptable variable chosen. Six features, namely HB, ruptured, D-dimer, PLT, sex, and WBC, were entered before the λ1se line

LASSO was performed to decrease the model’s complexity and reduce redundant or irrelevant data in the training group. Six variables, namely HB, ruptured aneurysm, D-dimer, PLT, sex, and WBC, were entered before λ1se and were selected for model establishment (Fig. 2A and B).

**Fig. 3:**
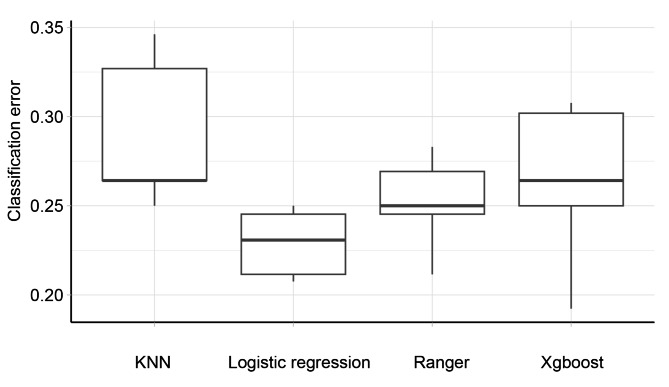
Classification error for evaluating the discrimination power of various machine learning algorithms. The classification values for intraoperative blood transfusion were 0.2903 of the K-nearest neighbor, 0.2290 of logistic regression, 0.2518 of ranger, and 0.2632 of the extremely gradient boosting model, respectively

The balanced performance evaluation with nested resampling results in the training set showed that the CE was 0.2903 of the KNN, 0.2290 of logistic regression, 0.2518 of ranger, and 0.2632 of the extremely gradient boosting model, respectively, with no statistical difference between the four algorithms (P = 0.0738, F = 6.9406, Fig. 3). The CE, AUC, accuracy, and specificity for each machine learning method are depicted in Table 2.

**Table 2:** Performance evaluation of four machine learning algorithms

	CE	AUC	Accuracy	Specificity
**KNN**	0.2903	0.7653	0.7097	0.8788
**LR**	0.2290	0.7993	0.7710	0.8981
**Ranger**	0.2518	0.7896	0.7482	0.8998
**Xgboost**	0.2632	0.7583	0.7368	0.8577

AUC, Area under the curve; CE, Classification error; Xgboost, Extremely gradient boosting machine; KNN, K-nearest neighbor; LR, Logistic regression

### Nomogram establishment and assessment

**Fig. 4:**
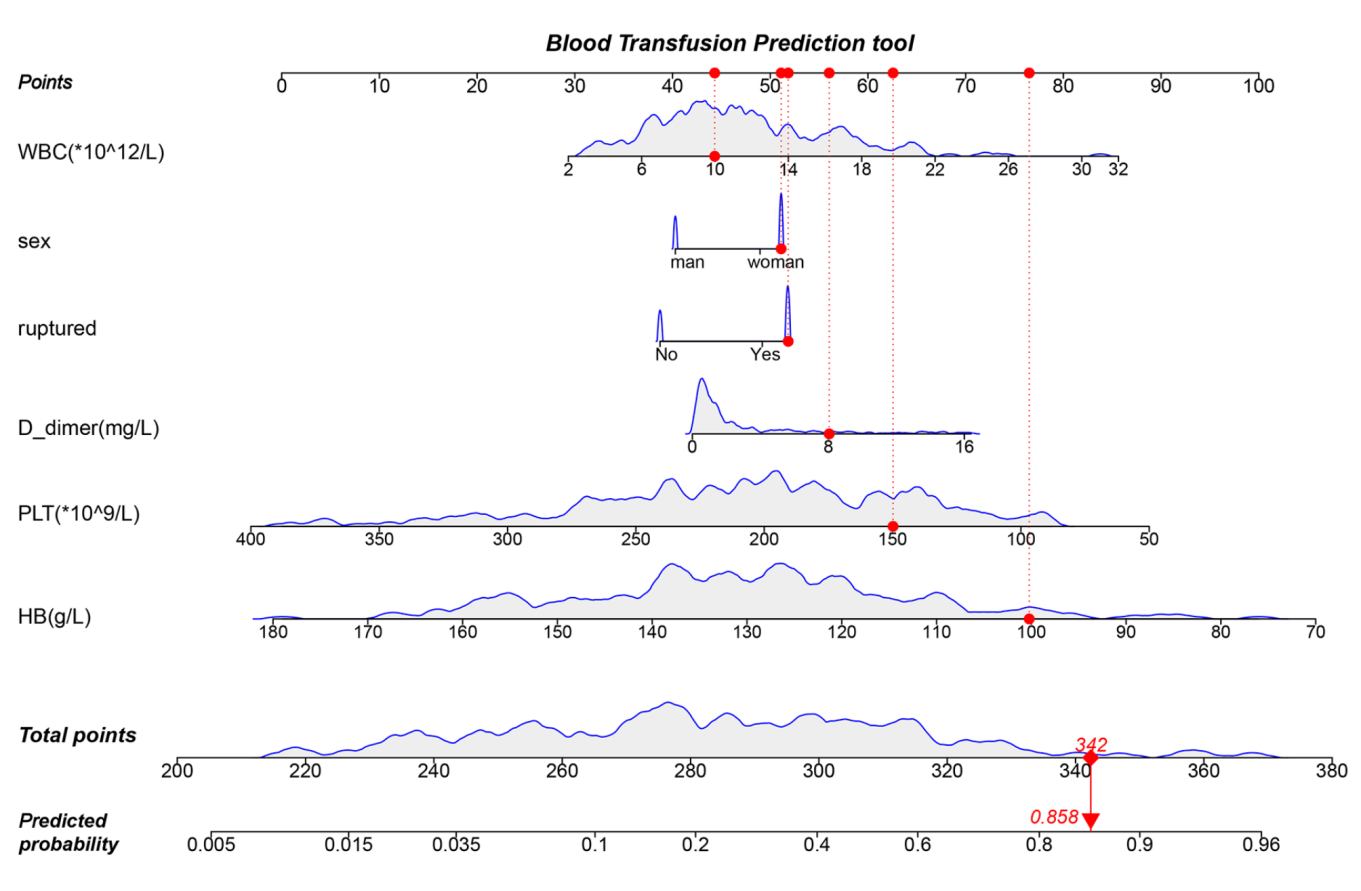
Nomogram to predict the probability of intraoperative blood transfusion in the patients who underwent intracranial aneurysm surgery. For example, a female patient with a ruptured aneurysm, D-dimer (8 mg/L), WBC (10 × 10^12^), PLT (150 × 10^9^), and HB (100 g/L), had a total score of 342 in terms of these variables and a predicted probability of intraoperative blood transfusion of 85.8%. WBC, White blood cell; HB, Hemoglobin; PLT, Platelet

**Fig. 5:**
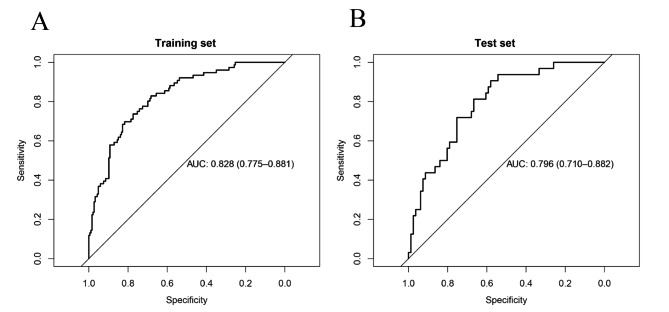
ROC curves for evaluating the discrimination power of the nomogram based on logistic regression algorithms. The AUC values for intraoperative blood transfusion were 0.828 (0.775, 0.881) and 0.796 (0.710, 0.882) in the development (A) and validation (B) groups, respectively

The LR model was used to establish the nomogram with the development data. The nomogram was established based on the six above variables to predict intraoperative blood transfusion in patients undergoing intracranial aneurysm clipping surgery (Fig. 4). The variables are shown in rows 2 to 7, in which the values are acquired from the patient. The first row is the point assigned to each variable’s measurement, and the assigned points for all of the variables are then summed, with the total can be located on the line of the total points. After locating the total points, a vertical line is drawn down to the bottom line to obtain the predicted probability of transfusion. The AUC values of the nomogram for intraoperative blood transfusion prediction were 0.828 (0.775, 0.881) and 0.796 (0.710, 0.882) in the development (Fig. 5A) and validation (Fig. 5B) groups, respectively.

## Discussion

In the present study, we identified six preoperative risk factors for intraoperative blood transfusion, namely HB, D-dimer, PLT, ruptured aneurysm, sex, and WBC. Then, we used the nested resampling method to evaluate the balanced performance of various machine learning algorithms with the six variables, and observed no statistical difference between the four models. We established a nomogram with the LR algorithm to predict the risk of intraoperative blood transfusion in aneurysm surgery. The performance of the established nomogram showed a good discriminative ability in both the development and validation sets.

The feature selection process with LASSO removes redundant or irrelevant features in the data with limited loss of information, while the fewer variables increase the interpretation and application of the model. The selection process of the shrinking ability of this operator depends on modifying the absolute value of the coefficient of functions. Features with non-zero coefficient values were chosen for analysis. Machine learning algorithms establish complex models and make accurate decisions when given relevant data. Some machine learning algorithms have been used within the anesthesiology and pain fields, such as for predicting mortality, and kidney injury after cardiac surgery [16, 17], predict postoperative pain, or identify the need for pain consults [11, 18]. These explorations are primarily focused on the postoperative period, while preoperative data acquisition for predicting intraoperative blood transfusion is infrequent [19]. In this study, we used four machine learning methods and acquired a balanced performance evaluation, with no statistical difference between the four models.

Considering the good interpretability and generalizability, we established a nomogram using the LR algorithm and performed an ROC curve analysis to assess the performance of the predictive model. The AUC in the development and validation groups was consistent with the machine learning results, indicating good performance of the model. Generally, the model used in this study offered a good screening method to calculate a patient’s probability of requiring intraoperative blood transfusion during intracranial aneurysm surgery. The benefit of predicting intraoperative blood transfusion may allow clinicians to prepare blood-saving strategies more adequately, tailor their surgery more carefully, and ultimately reduce the complications greatly.

The prediction nomogram for blood transfusion is useful when ordering allogeneic blood and preparing preoperative autologous blood preoperatively. The use of the nomogram could result in substantial savings by decreasing allogenic blood consummation. Patients with a high-risk score (≥ 0.5) to receive a perioperative blood transfusion should be encouraged to use the autologous blood donation strategy, which would minimize the possibility of an allogenic transfusion. Studies have shown that autologous blood predisposition before surgery is very effective in decreasing perioperative allogenic blood transfusion [20, 21]. Moreover, acute intraoperative hemodilution and salvage have been shown to significantly increase postoperative hemoglobin levels and improve patients’ outcomes [22]. Additionally, patients with a low preoperative HB level tend to have a high nomogram score and should be encouraged to take preoperative pharmacological treatments, such as erythropoietin or antifibrinolytic management, or intraoperative self-blood collection.

Traditionally, physicians decide to perform a blood transfusion largely based on HB levels [23–25]. However, some physicians have reported significant differences in the HB thresholds for transfusion [26, 27]. Therefore, we established a nomogram by combining patient characteristics other than serum HB levels. A particular strength of this study is that it accounted for a wide range of preoperative variables associated with the receipt of blood transfusions. PLT is a crucial constituent of clotting, and a low PLT count has been identified as an independent risk factor of intraoperative blood transfusion during abdominal aneurysm surgery [28]. Moreover, a PLT count < 130 × 10^9^/L has a 3.9-fold relative risk of transfusion when compared with a PLT > 130 × 10^9^/L. Each 10 × 10^9^/L increase in platelet count was associated with an 11% decrease in severe bleeding risk [29].

A high D-dimer level is another determinant for intraoperative blood transfusion during aneurysm surgery. D-dimer is an indicator of coagulation and fibrinolysis and is suggestive of a fibrinolysis profile in the body [30]. High D-dimer levels may be attributed to the following [31]: a ruptured aneurysm activating the coagulation system, resulting in coagulopathy and massive bleeding during surgery; and a ruptured aneurysm increasing intracranial pressure, increasing the duration and difficulty of the surgery. Further, our results suggest that WBC is another risk factor for intraoperative blood transfusion; this may be attributed to the systemic inflammatory response activating platelets, coagulation, and fibrinolysis, as well as up-regulating platelet adhesion receptors and increasing monocyte-platelet conjugates, all of which result in coagulation disorder and the consumption of constituents [32]. Lastly, our results identified that being female is a risk factor for perioperative blood transfusion.

The study has several limitations that should be considered when interpreting the present results. As with all retrospective studies, there may be some unknown confounders that were not referred to in the analysis. However, we collected key preoperative characteristics in an integrated online system, thereby minimizing the likelihood of selection bias.

## Conclusions

HB, platelet, D-dimer, sex, WBC, and aneurysm rupture before surgery are relevant factors for intraoperative blood transfusion during aneurysm surgery. Machine learning algorithms with nested resample methods result in a good performance evaluation. The described nomogram, using a logistic regression algorithm has a good discriminative ability to predict intraoperative blood transfusion during aneurysm surgery.

## Data Availability

The data that support the findings of this study are available from the corresponding author upon reasonable request.

## Abbreviations

APTT: Activated partial thromboplastin time
AUC: Area under the curve
ASA: American Society of Anesthesiologists
CE: Classification error
CV: Cross-validation
DM: Diabetes mellitus
xgboost: Extremely gradient boosting machine
HB: Hemoglobin
KNN: K-nearest neighbor algorithm
LASSO: Least absolute shrinkage and selection operator
LR: Logistic regression model
PLT: Platelet
PT: Prothrombin time
ROC: Receiver operating characteristic
TP: Total protein
ALB: Albumin
WBC: White blood cell
